# Expanding the conjugation toolbox of DNA-stabilized silver nanoclusters with biotin-streptavidin

**DOI:** 10.1039/d6ra09400g

**Published:** 2026-10-30

**Authors:** Vanessa Rück, Simon Wenzel Lind, Christian Brinch Mollerup, Tom Vosch

**Affiliations:** a Department of Chemistry, University of Copenhagen Universitetsparken 5 2100 Copenhagen Denmark tom@chem.ku.dk; b Department of Forensic Medicine, University of Copenhagen Frederik V's Vej 11 2100 Copenhagen Denmark

## Abstract

DNA-stabilized silver nanoclusters (DNA-AgNCs) combine molecular-scale dimensions with sequence-dependent optical properties, making them promising candidates for bioimaging applications. However, strategies for site-specific conjugation of DNA-AgNCs remain comparatively limited. Here, we expand the conjugation toolbox of DNA-AgNCs through biotin functionalization of previously developed single-stranded DNA-AgNC containing a [Ag_16_Cl_2_]^8+^ cluster. Incorporation of biotin either at 3′ end or in the middle of the sequence, enabled selective immobilization of the resulting biotinylated DNA-AgNCs on streptavidin-coated poly(methyl methacrylate) (PMMA) microparticles. Importantly, biotin functionalization and subsequent particle attachment largely preserved the characteristic optical properties and fluorescence lifetimes of the DNA-AgNCs, while control experiments showed negligible nonspecific binding of the non-biotinylated DNA-AgNCs. These results demonstrated that biotin–streptavidin interaction provides a robust strategy for the modular functionalization of DNA-AgNCs without compromising their photophysical properties, opening opportunities for their integration into future bioimaging and sensing applications.

## Abstract 2

Site-specific biotin functionalization of DNA-AgNCs enables streptavidin binding while largely preserving their structural integrity and optical properties, expanding the toolbox for DNA-AgNC conjugation.

## Graphical abstract

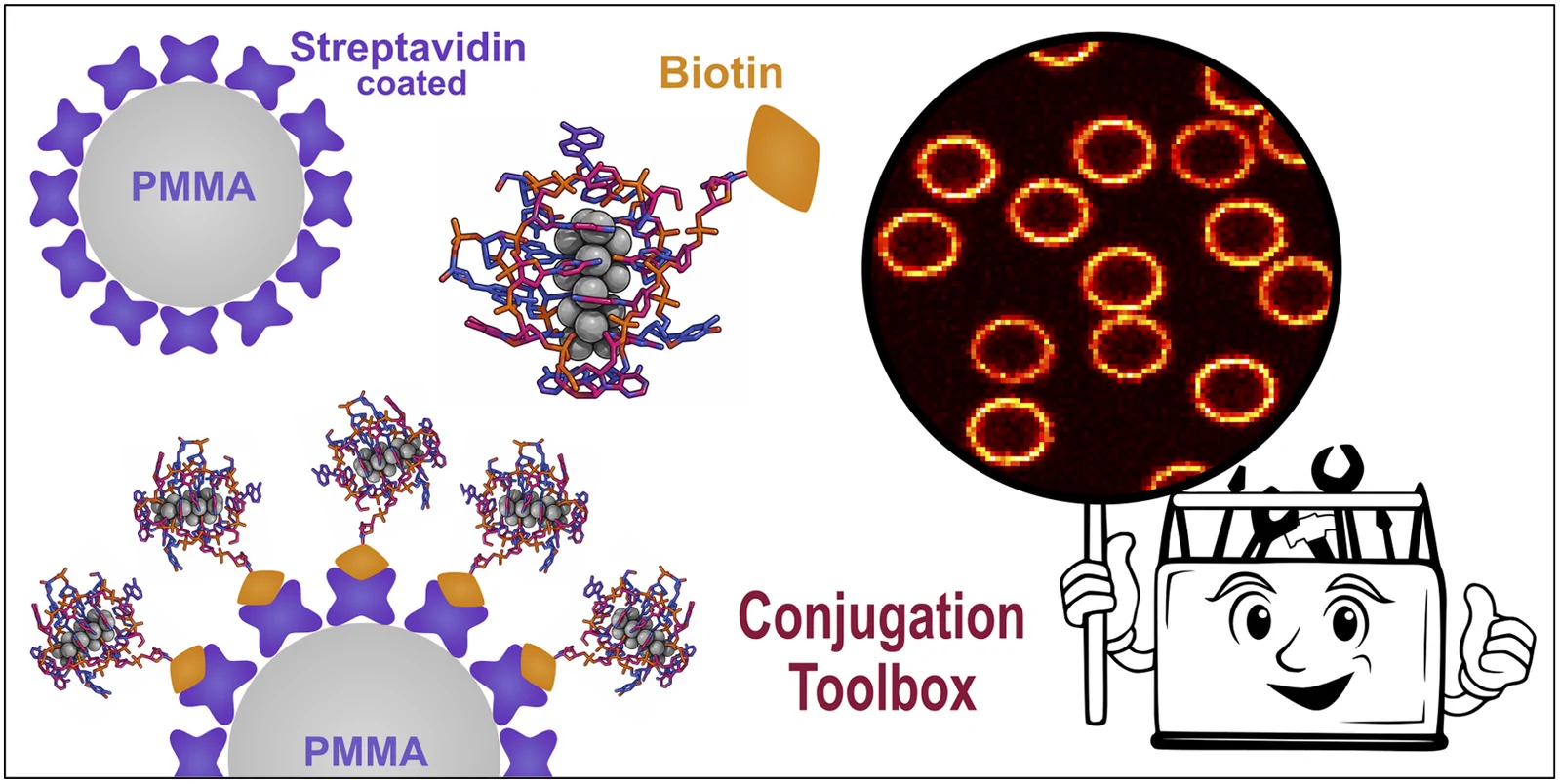

## Introduction

DNA-stabilized silver nanoclusters (DNA-AgNCs) have emerged as a versatile class of fluorescent nanomaterials that combine molecular-scale dimensions with tunable and sequence-dependent optical properties.^1–15^ These unique characteristics make DNA-AgNCs highly attractive for a wide range of applications, particularly in biosensing and bioimaging.^16,17^ The development of such applications, however, often requires the conjugation of DNA-AgNCs to biomolecules or other materials. Achieving this requires robust and reliable conjugation strategies that preserve the structural integrity and fluorescence properties of the DNA-AgNCs while allowing precise control over the attachment site.

Despite growing interest in DNA-AgNCs, strategies for their site-specific conjugation remain limited. To date, click chemistry has been reported for the conjugation of HPLC-purified DNA-AgNCs; however, the reported approach did not enable single-site functionalization of the nanoclusters.^18^ This highlights the need to investigate additional conjugation strategies that enable precise and controlled functionalization of DNA-AgNCs. In particular, evaluating the compatibility of established bioconjugation methods with DNA-AgNCs is an important step toward expanding their functional versatility and facilitating their integration into more complex molecular systems.

Among the various reported DNA-AgNCs, DNA_2_-[Ag_16_Cl_2_]^8+^ has received particular attention due to its resolved atomic structure, near-infrared emission, large Stokes shift and stability towards chloride ions.^6,19–23^ Recent work demonstrated that rational sequence design, specifically the incorporation of thymine spacers, enabled the conversion of the original two oligomer scaffold into single stranded analogues while preserving both the AgNC integrity and optical behavior.^24^

The ability to generate single-stranded versions of these DNA-AgNCs opens new opportunities for site-specific functionalization. In particular, the introduction of functional groups at defined positions along the DNA backbone allows for controlled conjugation to biomolecules and surfaces. Among the available bioconjugation strategies, biotin-streptavidin coupling is particularly attractive because it relies on one of the strongest known non-covalent interactions in nature. This interaction combines ultrahigh affinity and specificity with compatibility across a wide range of experimental conditions.^25,26^ These properties have established biotin-streptavidin coupling as an important tool in bioassays, imaging, and nanobiotechnology, enabling reliable immobilization and targeting of functional probes. So far, only limited attempts have been made to introduce the biotin-streptavidin binding strategy to DNA-AgNCs, mainly using unpurified DNA-AgNCs of unknown structure.^27–29^

In this work, we build upon our previously developed single-stranded versions of DNA_2_-[Ag_16_Cl_2_]^8+^ by introducing biotin groups positioned either at the 3′ terminus or within the thymine section of the DNA sequence. The resulting biotinylated DNA-AgNCs retain the characteristic optical properties of the parent nanocluster. As a model system, we demonstrate efficient binding to streptavidin-coated poly(methyl methacrylate) (PMMA) beads, providing clear evidence of successful functionalization.

Our results establish that the well-known biotin-streptavidin coupling strategy can be extended to DNA-AgNCs without compromising their properties. The use of the single-stranded version of DNA_2_-[Ag_16_Cl_2_]^8+^ overcomes the challenge of multiple functionalization sites associated with the original double-stranded version.^18^ This advancement represents an important step toward integrating DNA-AgNCs into biological systems and highlights their potential as fluorescent probes for future applications.

## Results and discussion

### Synthesis

In our previous work,^24^ we reported the successful creation of three single-stranded version of the well characterized near-infrared emitting DNA_2_-[Ag_16_Cl_2_]^8+^.^19,20^ This was achieved by adding a four to six thymine segment in between two 5′-CACCTAGCGA-3′ segments, referred to as 2xDNA(T_4_), 2xDNA(T_5_) and 2xDNA(T_6_). We demonstrated that the photophysical properties and structure of the [Ag_16_Cl_2_]^8+^ cluster remained largely unchanged.^24^ Here we engineered two biotinylated versions; one based on the 2xDNA(T_6_)-[Ag_16_Cl_2_]^8+^ where we attach a biotin group on the 3′ end (2xDNA(T_6_)-B-[Ag_16_Cl_2_]^8+^) and one based on the 2xDNA(T_5_)-[Ag_16_Cl_2_]^8+^ where we exchanged the middle thymine of the T_5_ segment with a biotin-modified thymine (2xDNA(TT-B-TT)-[Ag_16_Cl_2_]^8+^). Synthesis of DNA-AgNCs using the modified biotinylated sequence was performed as previously reported.^24^ Briefly, the hydrated oligomer strands were mixed with AgNO_3_ in 10 mM ammonium acetate (NH_4_OAc) and 30 mM sodium chloride (NaCl), and a freshly prepared NaBH_4_ solution was added after an incubation time of 15 min to promote AgNC formation. The final ratio between the components was [DNA] : [AgNO_3_] : [NaBH_4_] = 25 µM : 187.5 µM : 93.75 µM. After 7 days at 4 °C, the mixture was purified by high-performance liquid chromatography (HPLC), yielding successful separation. Details on the HPLC purification and the corresponding chromatograms can be found in the SI (Fig. S1 and S2).

### Photophysical properties

The spectroscopic characterization of the purified 2xDNA(T_6_)-B-[Ag_16_Cl_2_]^8+^ and 2xDNA(TT-B-TT)-[Ag_16_Cl_2_]^8+^ compounds shows that the photophysical properties remain nearly unaltered, see Fig. 1 and Table 1. The absorption maximum remains around 524 nm and the emission maxima are centered at around 750 nm. The average fluorescence decay times are also very similar and seem not to be affected by the biotin-group (see Table 1). Time-resolved fluorescence anisotropy measurements were used to determine the hydrodynamic volume. Compared to 2xDNA(T_5_)-[Ag_16_Cl_2_]^8+^ and 2xDNA(T_6_)-[Ag_16_Cl_2_]^8+^, the hydrodynamic volume increases in both cases as expected due to the additional biotin group.^30^

**Fig. 1:**
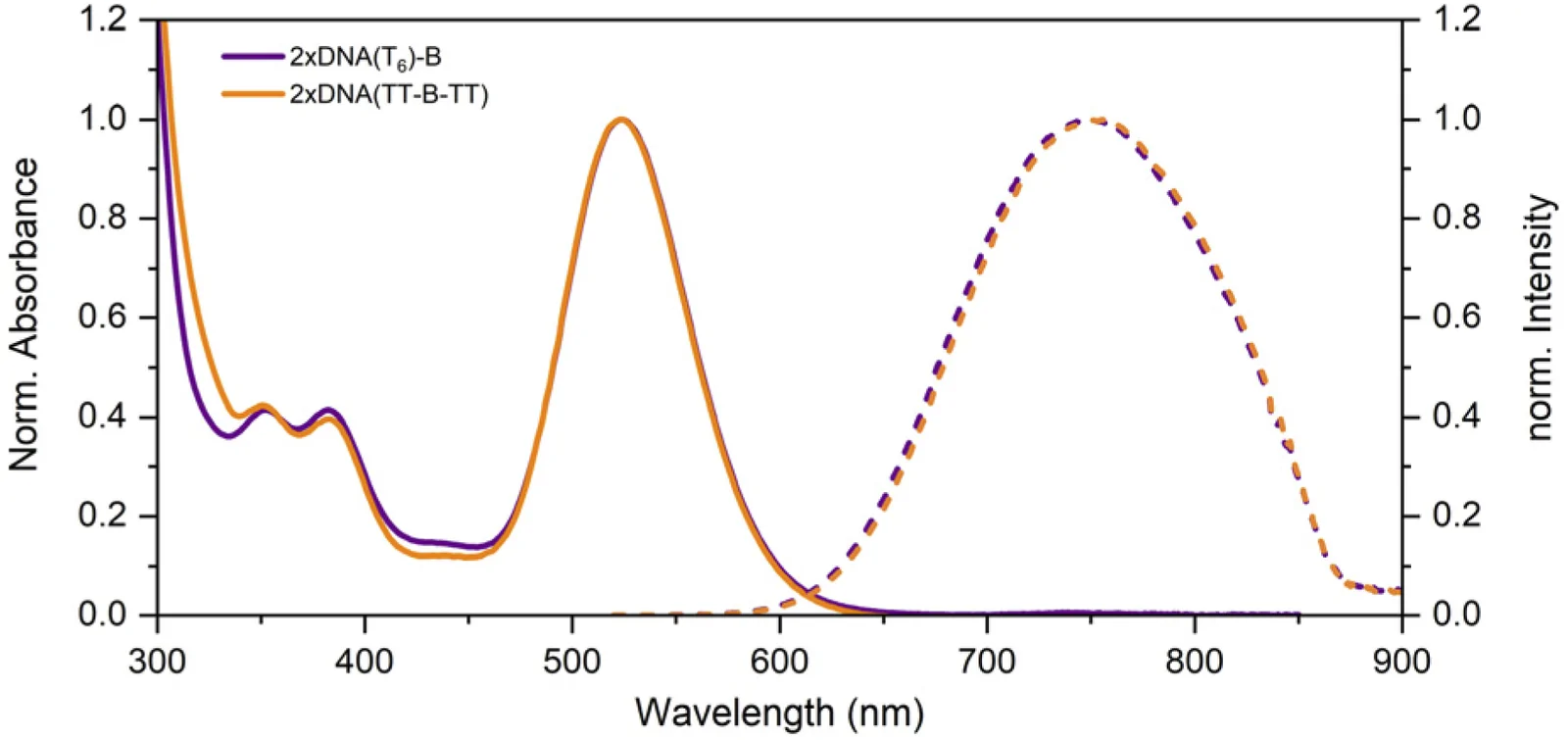
Normalized absorption and emission spectra of 2xDNA(T_6_)-B-[Ag_16_Cl_2_]^8+^ (purple) and 2xDNA(TT-B-TT)-[Ag_16_Cl_2_]^8+^ (orange) in 10 mM NH_4_OAc at room temperature.

**Table 1:** Photophysical properties of 2xDNA(T_6_)-B-[Ag_16_Cl_2_]^8+^ and 2xDNA(TT-B-TT)-[Ag_16_Cl_2_]^8+^ compared with the non-biotinylated versions 2xDNA(T_5_)-[Ag_16_Cl_2_]^8+^ and 2xDNA(T_6_)-[Ag_16_Cl_2_]^8+^

Sample	Abs (nm)	Em (nm)	*τ* (ns)	*Φ* (ns)	*V*_hydro_ (nm^3^)
2xDNA(T_5_)^a^	524	750	3.46	3.45	15.97
2xDNA(TT-B-TT)	523	752	3.46	3.87	17.92
2xDNA(T_6_)^a^	523	747	3.44	3.67	16.99
2xDNA(T_6_)-B	524	750	3.43	4.12	19.07

a Data taken from Rück *et al.*^24^

### Compositional analysis

Additionally, the biotinylated DNA-AgNCs were analysed by electrospray ionization mass spectrometry (ESI-MS) to verify whether biotin modification affects the composition of the AgNC. The mass spectra of both biotinylated variants are shown in Fig. 2, S5 and S6. The AgNC charge were assigned manually (see also SI) using a procedure similar to the one reported recently by Lin *et al.*^31^ The experimental mass data contains a compound comprising one DNA strand, two chlorido ligands, and 16 silver atoms (*N* = 16) with *N*_0_ = 6, corresponding to the expected composition. Notably, the most intense peak corresponds to a species containing 17 silver atoms (*N* = 17). Given the fact the photophysical properties are unchanged, we can exclude that this extra silver cation binds to the [Ag_16_Cl_2_]^8+^ core. Instead, we suggest that the extra silver cation is coordinated to the biotin moiety. Previously, we also found indications of coordination of Ag^+^ to the bicyclononyne (BCN) conjugation sites.^18^ A minor peak corresponding to a species with 18 silver atoms (*N* = 18) is also observed. The original DNA_2_-[Ag_16_Cl_2_]^8+^ also exhibited a small Ag^+^ adduct peak,^18^ were an additional Ag^+^ could be bound to the terminal adenine.^19^ Hence, the *N* = 18 peak can analogously be explained as an Ag^+^ adduct of the *N* = 17 peak. Overall, these mass spectrometry results are consistent with the spectroscopic characterization and further support the preservation of the AgNC core upon biotinylation.

**Fig. 2:**
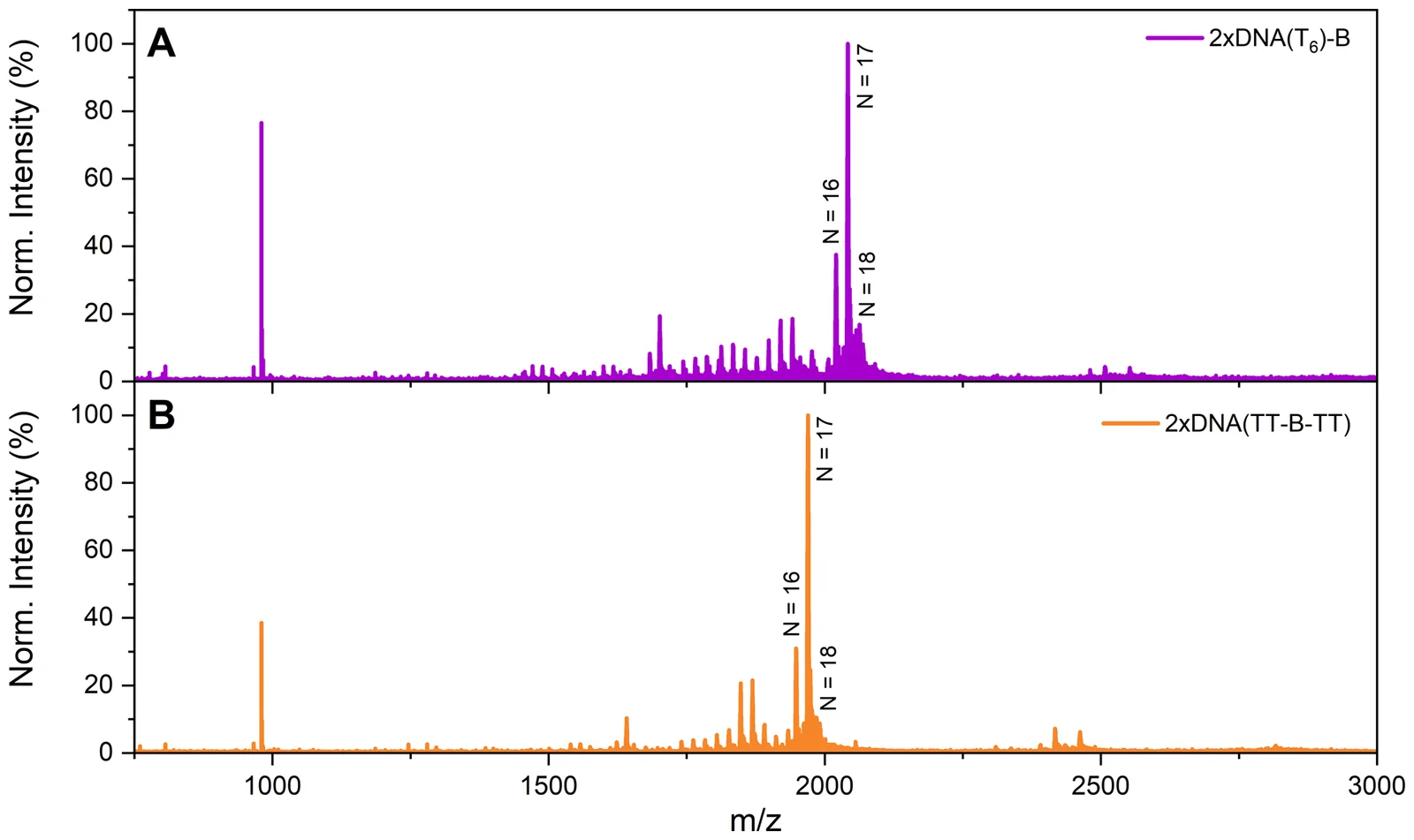
Mass spectra (A) 2xDNA(T_6_)-B-[Ag_16_Cl_2_]^8+^ and (B) 2xDNA(TT-B-TT)-[Ag_16_Cl_2_]^8+^ in 10 mM NH_4_OAc at room temperature. N indicates the total number of Ag atoms. *N*_0_ (number of Ag^0^ atoms) is 6 for *N* = 16, 17 and 18. See Fig. S7 and S8 for zooms of the *N* = 16, 17 and 18 peaks.

### Imaging and conjugation verification

To evaluate whether the introduced biotin functionality remains accessible for streptavidin binding, streptavidin-coated poly(methyl methacrylate) (PMMA) microparticles were employed as a model system. The 9 µm-sized PMMA spheres were incubated with either 2xDNA(T_6_)-B-[Ag_16_Cl_2_]^8+^ or 2xDNA(TT-B-TT)-[Ag_16_Cl_2_]^8+^ in 10 mM NH_4_OAc for 30 min to evaluate the capability for bioconjugation. As shown in Fig. 3A and D, homogeneous fluorescence, localized at the surface of the PMMA spheres was observed, confirming successful binding of both 2xDNA(T_6_)-B-[Ag_16_Cl_2_]^8+^ and 2xDNA(TT-B-TT)-[Ag_16_Cl_2_]^8+^ to the streptavidin-coated PMMA spheres. No significant differences in labeling efficiency or emission characteristics were observed between the 2xDNA(T_6_)-B-[Ag_16_Cl_2_]^8+^ and 2xDNA(TT-B-TT)-[Ag_16_Cl_2_]^8+^, indicating that both functionalization strategies provide efficient biotin access for streptavidin binding. This also demonstrated that suggested binding of Ag^+^ to the biotin (from the mass-spectrometry data), does not prevent the intended interaction of biotin with streptavidin. To confirm that the detected fluorescence originated from the DNA-AgNCs rather than from auto-fluorescence of the PMMA spheres, emission spectra were recorded after labeling. Fig. 3B and E display the characteristic Gaussian-like emission centered at approximately 732 nm and 752 nm (see for comparison Fig. 1 for the solution spectra) upon conjugation to the streptavidin-coated PMMA spheres. This indicates that the emission spectra are nearly unaltered for 2xDNA(TT-B-TT)-[Ag_16_Cl_2_]^8+^, while a small blue-shift is present for 2xDNA(T_6_)-B-[Ag_16_Cl_2_]^8+^. In addition, fluorescence lifetime measurements of the particle-bound biotinylated DNA-AgNCs revealed only a slight decrease in the intensity averaged decay time 〈*τ*〉 that may originate from the conjugation to the particle surface. To exclude nonspecific binding of the DNA-AgNCs, control experiments were performed using non-biotinylated 2xDNA(T_6_)-[Ag_16_Cl_2_]^8+^ under otherwise identical conditions, as well as streptavidin-coated PMMA spheres on their own in the absence of DNA-AgNCs. As shown in Fig. S7 and S8, both samples exhibited only low auto-fluorescence signals. In particular, the streptavidin-coated PMMA spheres incubated with 2xDNA(T_6_)-[Ag_16_Cl_2_]^8+^ for 30 minutes and subsequently washed following the same procedure showed no significant fluorescence signal, confirming that 2xDNA(T_6_)-[Ag_16_Cl_2_]^8+^ does not undergo substantial nonspecific binding to the streptavidin-coated PMMA spheres.

**Fig. 3:**
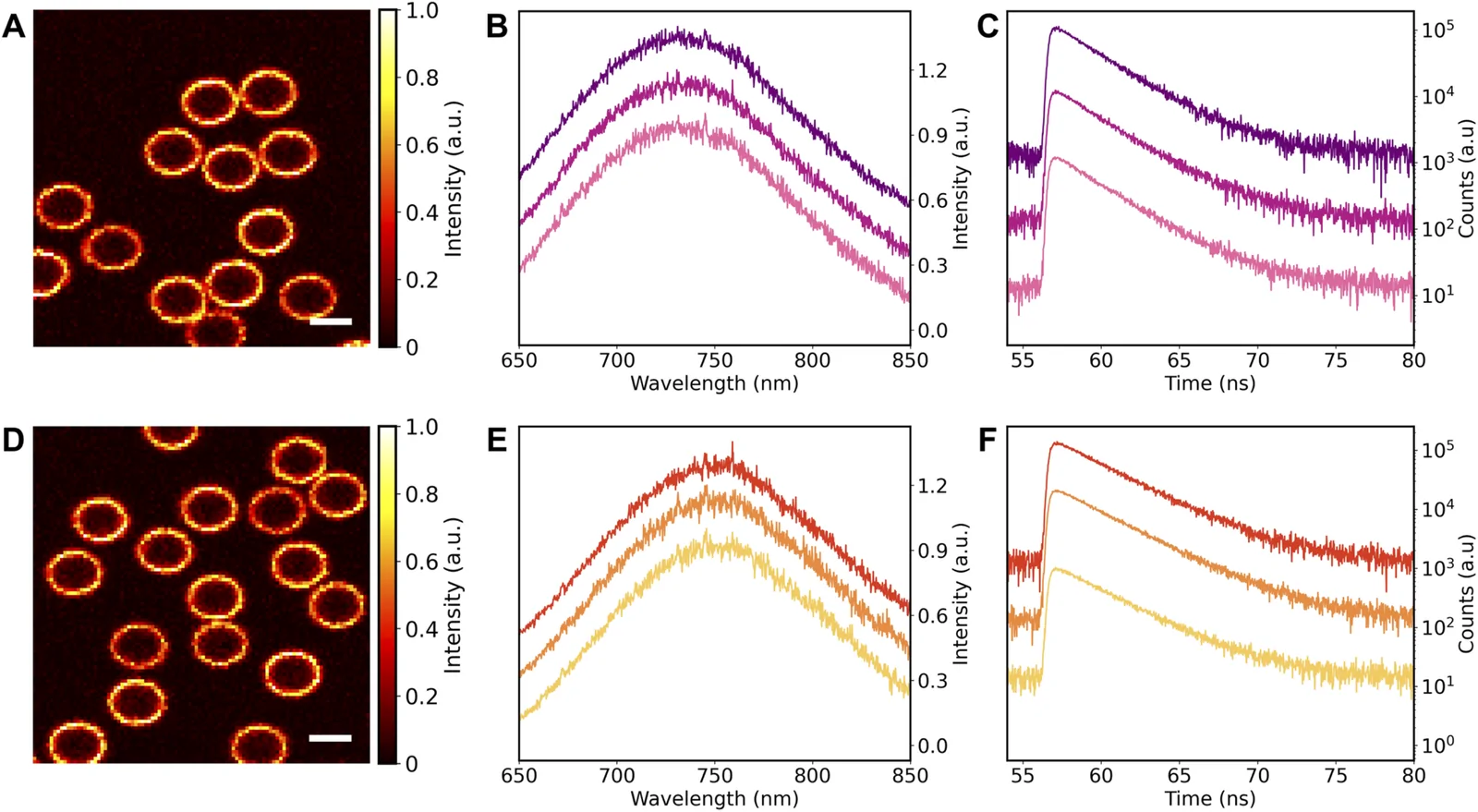
Binding of biotinylated DNA-AgNCs to streptavidin-coated PMMA microspheres. (A) Confocal fluorescence microscopy image of PMMA microspheres labeled with 2xDNA(T_6_)-B-[Ag_16_Cl_2_]^8+^ (scale bar: 10 µm), (B) emission spectra with emission maxima at 732, 734 and 731 nm, and (C) fluorescence decays with 〈*τ*〉 of 2.89, 2.88 and 2.89 ns. (D) Confocal fluorescence image of PMMA microspheres labeled with 2xDNA(TT-B-TT)-[Ag_16_Cl_2_]^8+^ (scale bar: 10 µm), (E) emission spectra with emission maxima at 753, 752 and 750 nm, and (F) fluorescence decays with 〈*τ*〉 of 3.18, 3.18 and 3.17 ns obtained.

## Conclusions

In conclusion, we demonstrate that the previously developed single-stranded version of DNA_2_-[Ag_16_Cl_2_]^8+^ can be successfully functionalized with biotin groups positioned either at the 3′ terminus or within the thymine-rich region without compromising the structural integrity or optical properties of the nanocluster. Mass spectrometric characterization confirmed that both biotinylated versions retain the atomically precise composition of the parent DNA_2_[Ag_16_Cl_2_]^8+^ system, while single PMMA sphere spectroscopy showed that the emission spectra and fluorescence decay time displayed only minor changes upon conjugation and surface attachment. The streptavidin-coated PMMA spheres are an ideal test platform for evaluating specific binding and evaluating labeling efficiencies. Comparable labeling behavior was observed for both 2xDNA(T_6_)-B-[Ag_16_Cl_2_]^8+^ or 2xDNA(TT-B-TT)-[Ag_16_Cl_2_]^8+^, and control experiments confirmed negligible nonspecific adsorption of the 2xDNA(T_6_)-[Ag_16_Cl_2_]^8+^ on the streptavidin-coated surface. Together, these findings establish biotin–streptavidin coupling as an effective strategy to expand the biofunctional toolbox of DNA-AgNCs.

## Conflicts of interest

There are no conflicts to declare.

## Supplementary Material

RA-OLF-D6RA09400G-s001

## Data Availability

The data supporting this article have been included as part of the supplementary information (SI). Supplementary information is available. See DOI: https://doi.org/10.1039/d6ra09400g.
