# Supplementary material for: Expanding the conjugation toolbox of DNA-stabilized silver nanoclusters with biotin-streptavidin
Source: RSC Adv. 2026 Oct 30;16(49):67461–5. doi: 10.1039/d6ra09400g (PMC13722049; doi:10.1039/d6ra09400g)
Supplement: Supplementary file 1 [file RA-016-D6RA09400G-s001.pdf]

Supplementary Information (SI) for RSC Advances.
This journal is © The Royal Society of Chemistry

Supporting Information

Expanding the Conjugation Toolbox of DNA-stabilized Silver Nanoclusters with Biotin-Streptavidin

Vanessa Rück,[a] Simon Wenzel Lind,[a] Christian Brinch Mollerup,[b] and Tom Vosch [a,*]

a. *Department of Chemistry, University of Copenhagen, Universitetsparken 5, 2100 Copenhagen, Denmark.*
b. *Department of Forensic Medicine, University of Copenhagen, Frederik V's Vej 11, 2100 Copenhagen, Denmark.*

Synthesis of 2xDNA-Biotin-AgNC

Biotinylated DNA oligonucleotides were purchased from Integrated DNA Technologies, the corresponding sequences and modification codes are provided below. Hydrated DNA oligonucleotides were mixed with $AgNO_3$ (Sigma Aldrich, ≥ 99.99%) in an aqueous solution containing 30 mM NaCl and 10 mM NH_4OAc (Sigma Aldrich, ≥ 98%) at pH 7. After 15 minutes, freshly prepared $NaBH_4$ (Sigma Aldrich, ≥ 99.99%) was added to reduce the silver ions and induce nanocluster formation. The optimized component ratio, [DNA]:[$AgNO_3$]:[$NaBH_4$] = 25 μM: 187.5 μM: 93.75 μM, was used according to a previously published protocol.[1] Following synthesis, the samples were stored at 4 °C for 7 days prior to purification by high-performance liquid chromatography (HPLC). The purified fractions were subsequently solvent-exchanged into 10 mM NH_4OAc using spin filtration with 3 kDa cut-off membrane filters (Amicon Ultracel-3) to ensure long-term stability of the DNA-AgNCs.

List of used DNA sequences (5'-3'):

- 2xDNA(TT-B-TT): CAC CTA GCG ATT – **/iBiodT/** – TT CAC CTA GCG A
- 2xDNA(T_6)-B: CAC CTA GCG ATT TTT TCA CCT AGC GA – **/3Bio/**

IDT Modification code: /iBiodT/

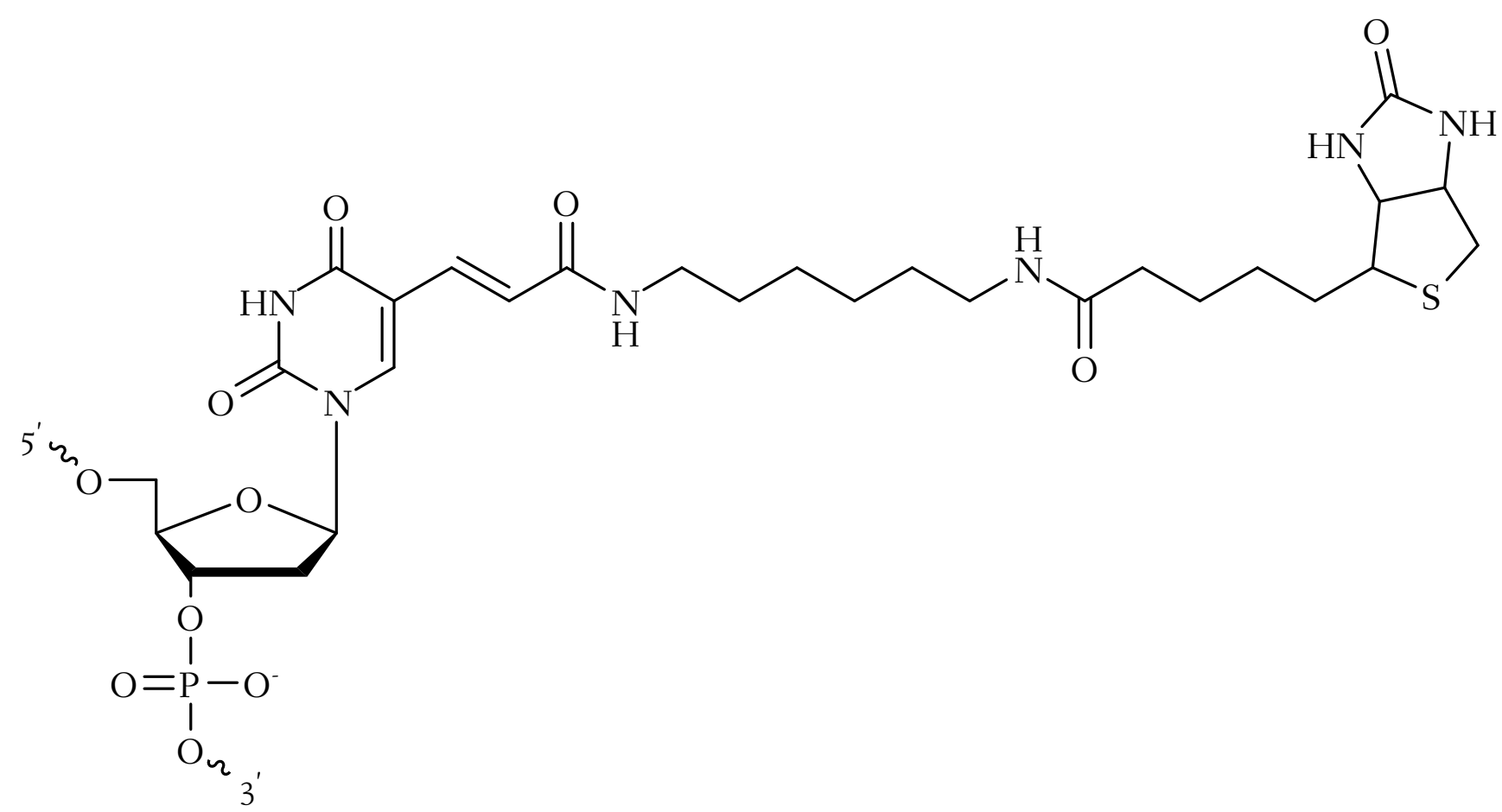

IDT Modification code: /3Bio/

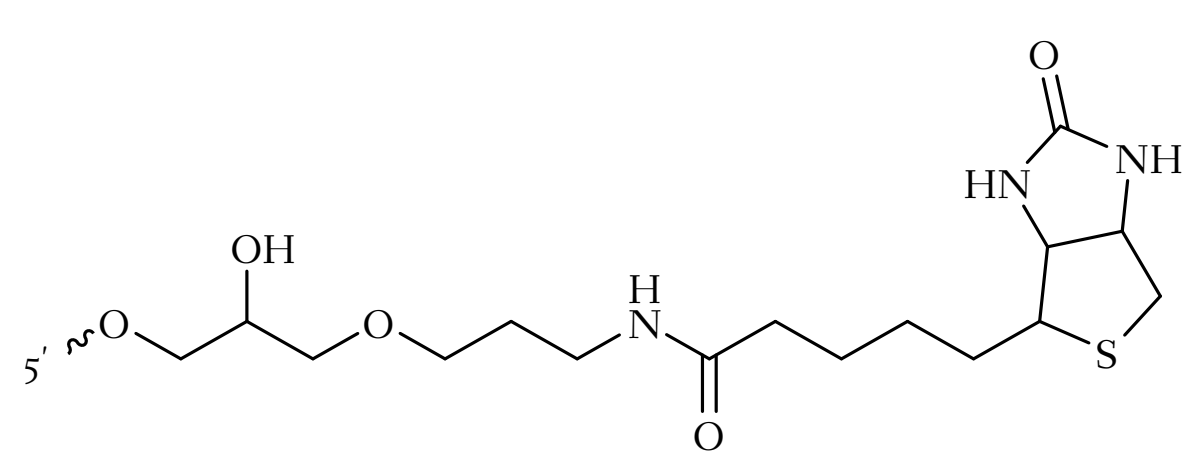

Other DNA sequences referenced in the manuscript (5’-3’):

- 2xDNA(T_4): CAC CTA GCG ATT TTC ACC TAG CGA
- 2xDNA(T_5): CAC CTA GCG ATT TTT CAC CTA GCG A
- 2xDNA(T_6): CAC CTA GCG ATT TTT TCA CCT AGC GA

HPLC purification of 2xDNA-Biotin-AgNC

The synthesis solution was upconcentrated until the sample displayed an intense red-brown color. An injection volume of 100–150 μL of the concentrated sample was used. HPLC purification was performed using a preparative HPLC system from Agilent Technologies with an Agilent Technologies 1100 Series UV-Vis detector, an Agilent Technologies 1260 Infinity fluorescence detector, and a Kinetex C18 column (5 μm, 100 Å, 250 × 4.6 mm), equipped with a fraction collector. The mobile phase was a gradient mixture of 35 mM triethylammonium acetate (TEAA, PanReac AppliChem) buffer in MilliQ water (A) and methanol (B). The flow rate was set to 1 mL/min. The HPLC method was carried out as follows: solvent B was maintained at 25% for the first 2 minutes, then increased linearly to 55% over the next 30 minutes (ΔB = 1% B/min). Subsequently, the solvent B was raised to 95% within 3 minutes and maintained for an additional 5 minutes to ensure complete removal of residual sample and nanoparticles from the column.

Fractions were collected based on the absorbance signal at 530 nm, while the DNA absorbance at 260 nm and the silver nanoparticle absorbance at 450 nm were monitored simultaneously. The AgNC emission was monitored at 730 nm with excitation at 530 nm. The corresponding chromatograms are shown in Figures S1–S2.

Mass spectrometry

ESI-MS measurements were performed with a Revident LC/Q-TOF (Agilent), using Dual AJS ESI source with acquisition mode MS in negative ion mode. Spectra were collected from m/z 750 to 3200 at 1.25 spectra/s with 120 V Fragmentor, 65 V skimmer and 0 V collision energy in Profile mode with MS threshold at 0. Source parameters were 200 °C gas temp, 10 L/min drying gas, 20 psi nebulizer, sheath gas at 150 °C with 12 L/min flow, 1000 V nozzle voltage, 750 V octopole RF. The QTOF was calibrated using QTOF LC/MS ESI-X Tuning Mix (Agilent), which contained compounds for negative mode in the mass range of m/z 113 to 2834. Reference mass was not utilized. All samples were injected using a 1290 Infinity III LC system (Agilent) with a flow-through needle multisampler, with a flow of 0.05 mL/min 50 mM NH_4OAc buffer at pH 7 – MeOH (80:20) and using 5 μL injection volume. The system was operated using MassHunter Acquisition v12 (Agilent), and the final spectra were generated by averaging multiple profile spectra surrounding the apex of the observed peak using MassHunter Qualitative Analysis v12 (Agilent).

The recorded data were analyzed and fitted with the open-source software EnviPat Web[2] (https://www.envipat.eawag.ch/index.php), together with a self-written MATLAB script.

Table S1. *Gaussian fits for the experimentally measured mass spectra (x_o^{exp}) and the corresponding theoretical mass distributions (x_o^{th}), and the absolute error calculated as $x_o^{exp} - x_o^{th}$.*

Name	z	Chemical formula	Molecular weight	x_o^{th}	x_o^{exp}	Error
2xDNA(T_6)-B-$[Ag_{16}Cl_2]^{8+}$	5-	$C_{268}H_{349}N_{93}O_{163}P_{26}S[Ag_{16}Cl_2]^{8+}$	10115.33 g/mol	2020.428	2020.514	0.086
2xDNA(T_6)-B-$[Ag_{17}Cl_2]^{9+}$	5-	$C_{268}H_{349}N_{93}O_{163}P_{26}S[Ag_{17}Cl_2]^{9+}$	10223.20 g/mol	2041.800	2041.846	0.046
2xDNA(T_6)-B-$[Ag_{18}Cl_2]^{10+}$	5-	$C_{268}H_{349}N_{93}O_{163}P_{26}S[Ag_{18}Cl_2]^{10+}$	10331.07 g/mol	2063.173	2063.006	-0.167
2xDNA(TT-B-TT)-$[Ag_{16}Cl_2]^{8+}$	5-	$C_{260}H_{336}N_{92}O_{152}P_{24}S[Ag_{16}Cl_2]^{8+}$	9754.20 g/mol	1948.20	1948.257	0.056
2xDNA(TT-B-TT)-$[Ag_{17}Cl_2]^{9+}$	5-	$C_{260}H_{336}N_{92}O_{152}P_{24}S[Ag_{17}Cl_2]^{9+}$	9862.06 g/mol	1969.574	1969.640	0.066
2xDNA(TT-B-TT)-$[Ag_{18}Cl_2]^{10+}$	5-	$C_{260}H_{336}N_{92}O_{152}P_{24}S[Ag_{18}Cl_2]^{10+}$	9969.93 g/mol	1990.946	1990.646	-0.3

Absorption measurements:

Absorption spectra were recorded using an Agilent Technologies Cary 3500 Flexible UV–Vis spectrophotometer equipped with a xenon flash lamp for visible and near-infrared measurements. Spectra were acquired in single-beam mode with baseline correction applied, and each spectrum was corrected by subtraction of the corresponding blank spectrum.

Steady-state emission measurements:

Steady-state fluorescence measurements were performed using a FluoTime300 instrument (PicoQuant). The fluorescence spectra were obtained by exciting the samples with a vertically-polarized 507.5 nm (LDH-PC-510, PicoQuant) picosecond-pulsed laser. All emission spectra have been corrected for the wavelength dependency of the detector.

Excitation spectra were recorded with a QuantaMaster 400 Fluorometer from Horiba equipped with a Xenon arc lamp, monitoring the emission at 750 nm. The spectra were corrected for the wavelength dependency of the detector and for the wavelength-dependent intensity difference of the excitation lamp.

Time-correlated single-photon counting (TCSPC) measurements:

Time-resolved fluorescence measurements were carried out using a PicoQuant FluoTime300 system equipped with a vertically polarized pulsed laser operating at 507.5 nm (LDH-PC-510, PicoQuant) as the excitation source. An integration time of 10 s was used to obtain at least 10,000 counts at the emission maximum at 750 nm. Data analysis was performed using FluoFit v4.6 software (PicoQuant). Fluorescence decay curves were fitted using a multiexponential reconvolution model including the instrument response function (IRF). The amplitude (αᵢ) and

decay time (τᵢ) components were subsequently used to calculate the intensity-weighted average lifetime, ⟨τ⟩, reported in Table 1.

Additionally, time-resolved fluorescence anisotropy measurements were carried out by recording the parallel and perpendicular fluorescence decay components at 750 nm. The samples were excited at 507.5 nm using a vertically polarized laser (LDH-P-C-510). The anisotropy data were fitted using a multiexponential reconvolution model for the fluorescence decay and a monoexponential model for the rotational correlation time (θ), including the IRF. The hydrodynamic volume (V_{hydro}) was subsequently calculated using the following equation:

$$\square = \frac{\square\square V_{hydro}}{k_B \, \square \, T}$$

where η is the dynamic viscosity of the solvent, T is the absolute temperature and k_B is the Boltzmann constant.

Imaging on home-build microscope:

Emission spectra, decay time measurements and confocal imaging was all performed on our home-built confocal microscope (Olympus IX71) in conjunction with a piezo scanning stage (Physike Instrumente, P-517.3CL). A continuum white-light laser (SuperK EXTREME EXB-6, NKT Photonics) was used as excitation source, delivering 514 nm laser light by sending the continuum output through an acousto-optic tunable filter (SuperK SELECT, NKT Photonics). The output laser was expanded and collimated by a lens system and cleaned by a 510 nm fluorescence filter (FF02-510/10-25, Semrock) in the excitation path before it was reflected by a 30:70 beam splitter (XF122, Omega Optical) and sent through an oil immersion objective (100x, NA = 1.4, Olympus). The objective collecting fluorescence light was directed through a 100 μm pinhole and the emission light was cleaned with two long pass filters, the first being a 514 nm laser line filter (LL01-514-25, Semrock) and the second being 633 nm long pass filter (LP02-633RE-25) to block the excitation beam. When measuring the emission spectra, the excitation source was tuned to a repetition rate of 77.7MHz, and the fluorescence was sent through a spectrograph (SP 2356 spectrometer, 300grooves/mm, Acton Research) onto a nitrogen cooled CCD camera (SPEC-10:100B/LN-eXcelon, Princeton Instruments) to collect the signal. For each spectrum, the piezo stage was set to scan over a 100 pixel by 100 pixel grid extending over an 80000 nm by 80000 nm area. The pixel dwell time was set to 2 ms. The emission spectra were wavelength and intensity corrected as reported previously.[3] For the confocal imaging the signal was directed to an avalanche photodiode (PerkinElmer, CD3226) and similar settings were used, except the pixel dwell time was set to 5 ms and the laser repetition rate was lowered to 11.1 MHz, so the fluorescence decay curve could be collected.

Sample preparation for confocal imaging

For the imaging experiments, 5 μL of streptavidin-coated poly(methyl methacrylate) (PMMA) beads (CD nanoparticles DNP-PA052, diameter: 9 μm) were added to a Lab-Tek chambered coverglass and incubated with 2 μL of biotinylated DNA-AgNCs in 100 μL of 10 mM NH₄OAc for 30 min at room temperature. Following incubation, all samples were washed three times with 200 μL of 10 mM NH₄OAc before measurements. Control experiments were performed under identical conditions using either the PMMA beads alone or beads incubated with 2xDNA(T_6)-$[Ag_{16}Cl_2]^{8+}$ lacking the biotin modification (also 3 wash steps were performed here).

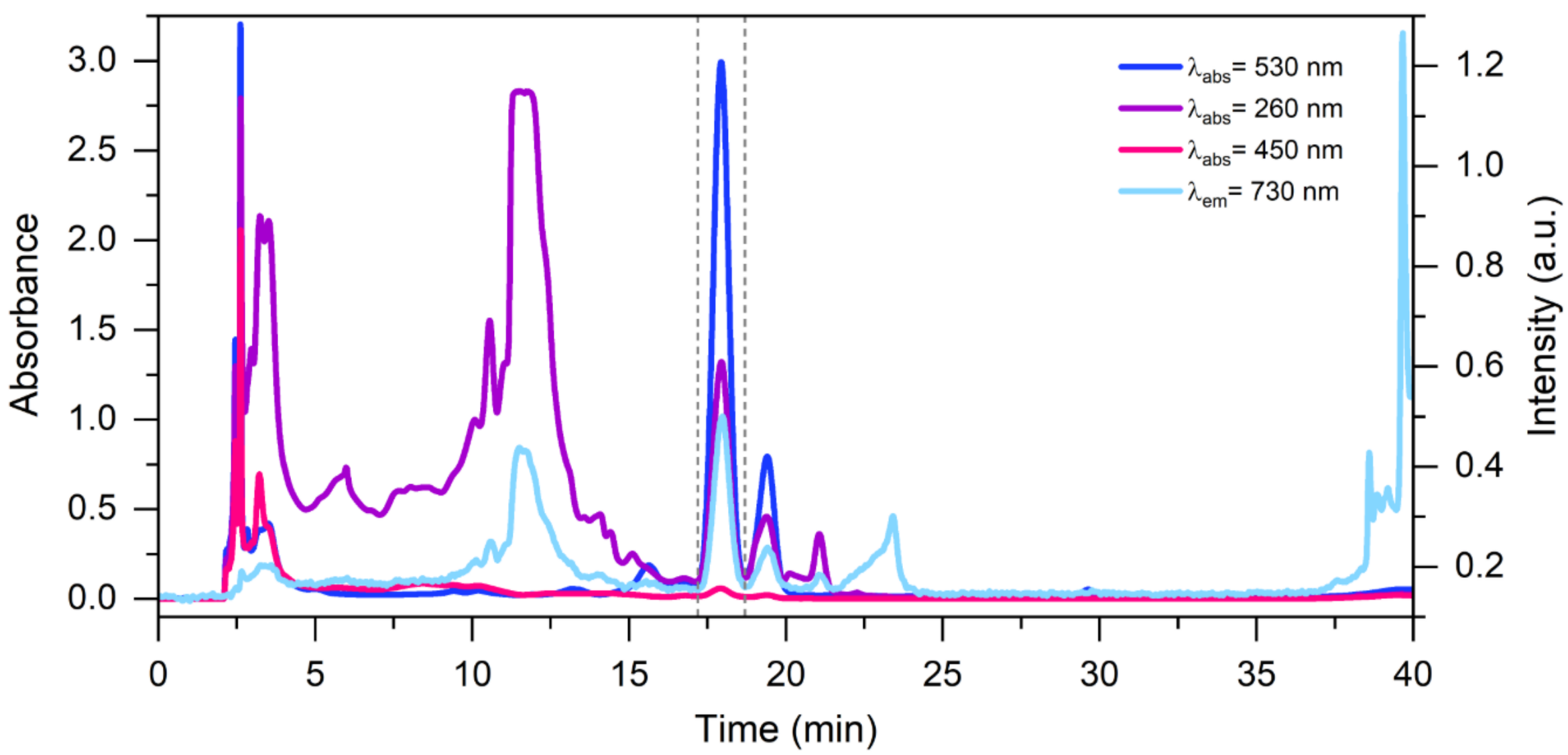

Figure S1: HPLC chromatograms of 2xDNA(T_6)-B-$[Ag_{16}Cl_2]^{8+}$ monitoring the absorption at 530 nm, 260 nm and 450 nm, and the fluorescence signal at 730 nm (exciting at 530 nm). The fraction was collected between 17.2 and 18.7 min (40.2-41.7 % B, dashed vertical lines).

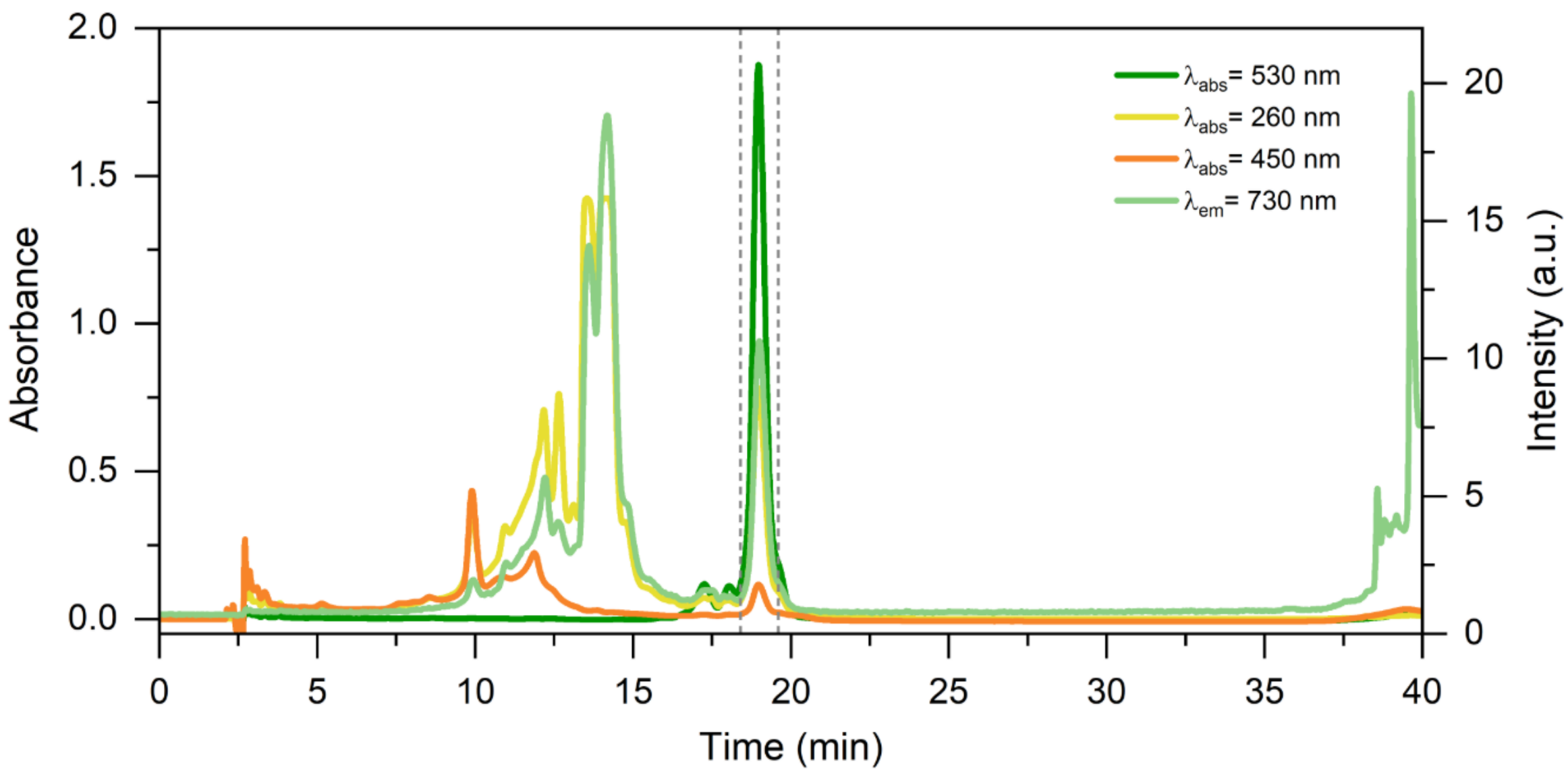

Figure S2: HPLC chromatograms of 2xDNA(TT-B-TT)-$[Ag_{16}Cl_2]^{8+}$ monitoring the absorption at 530 nm, 260 nm and 450 nm, and the fluorescence signal at 730 nm (exciting at 530 nm). The fraction was collected between 18.4 and 19.6 min (41.4-42.6 % B, dashed vertical lines).

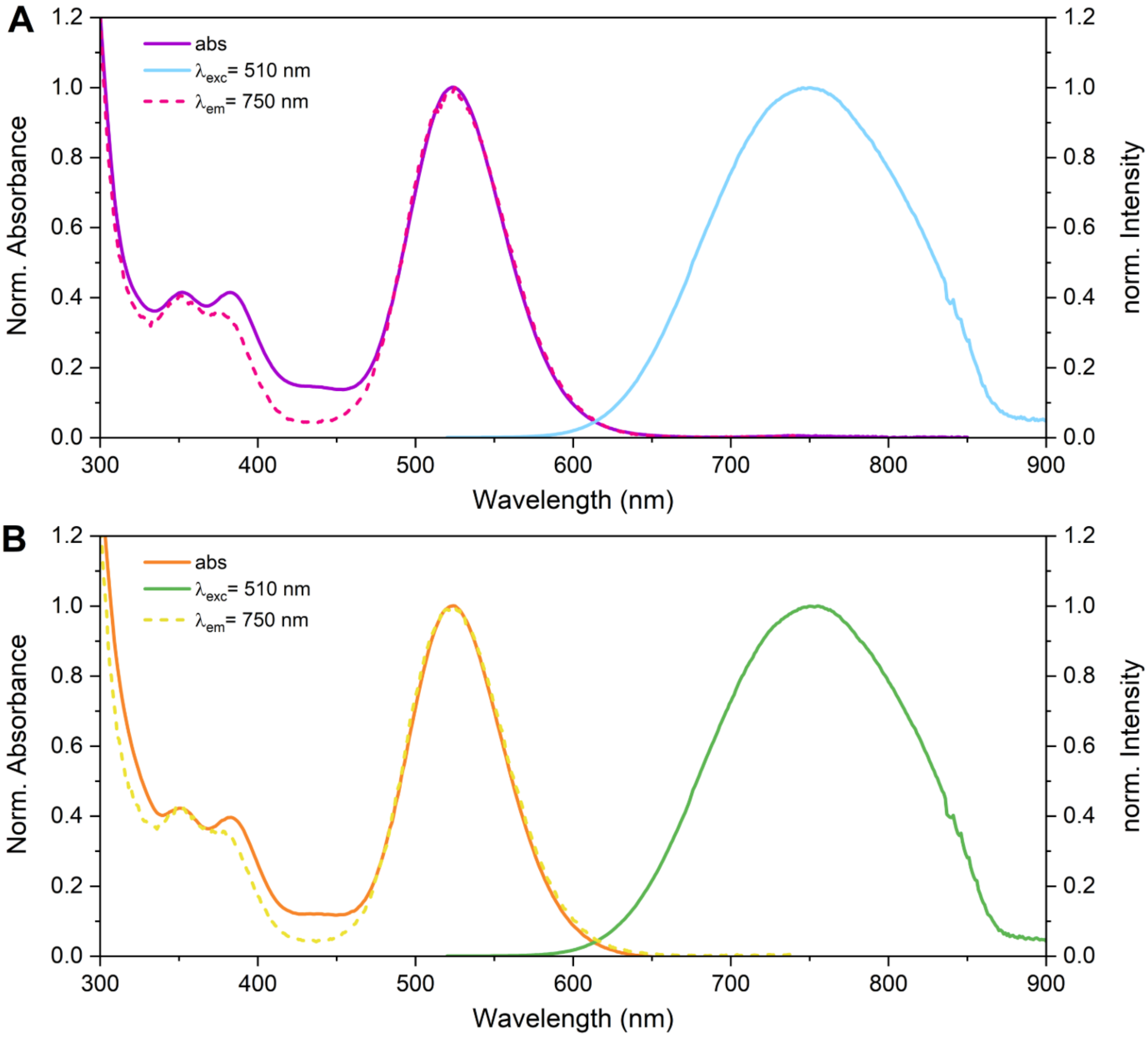

Figure S3: Normalized absorption, emission and excitation spectra of (A) 2xDNA(T_6)-B-$[Ag_{16}Cl_2]^{8+}$ and (B) 2xDNA(TT-B-TT)-$[Ag_{16}Cl_2]^{8+}$ in 10 mM NH_4OAc at room temperature.

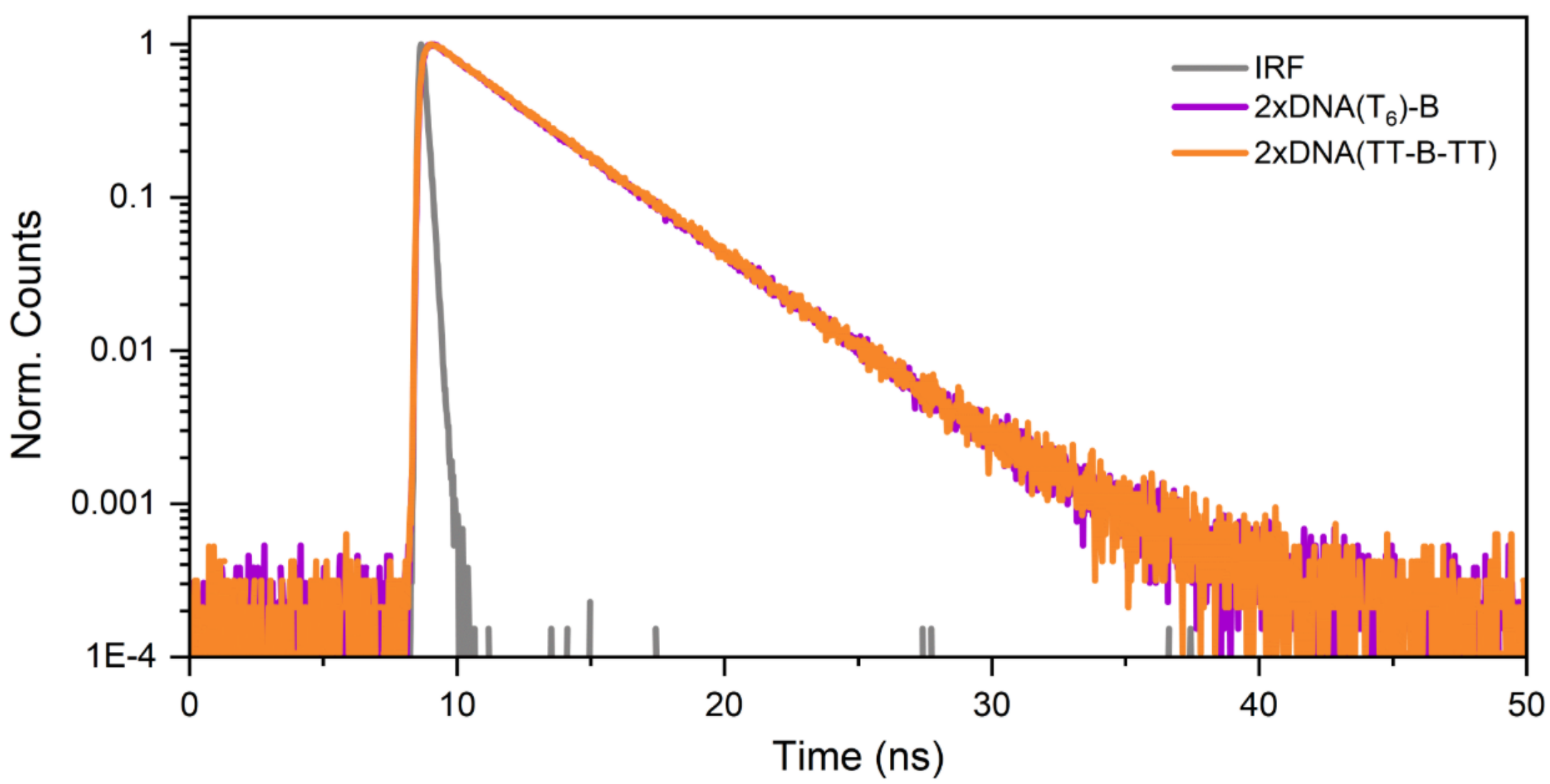

Figure S4: Fluorescence decays of 2xDNA(T_6)-B-$[Ag_{16}Cl_2]^{8+}$ and 2xDNA(TT-B-TT)-$[Ag_{16}Cl_2]^{8+}$ in 10 mM NH_4OAc measured at 740 nm (λ_{exc}= 507.5 nm) at room temperature. The decays were fitted with a bi-exponential reconvolution function, and the respective intensity-weighted average decay times are reported in Table 1. The dark gray trace is the instrument response function (IRF).

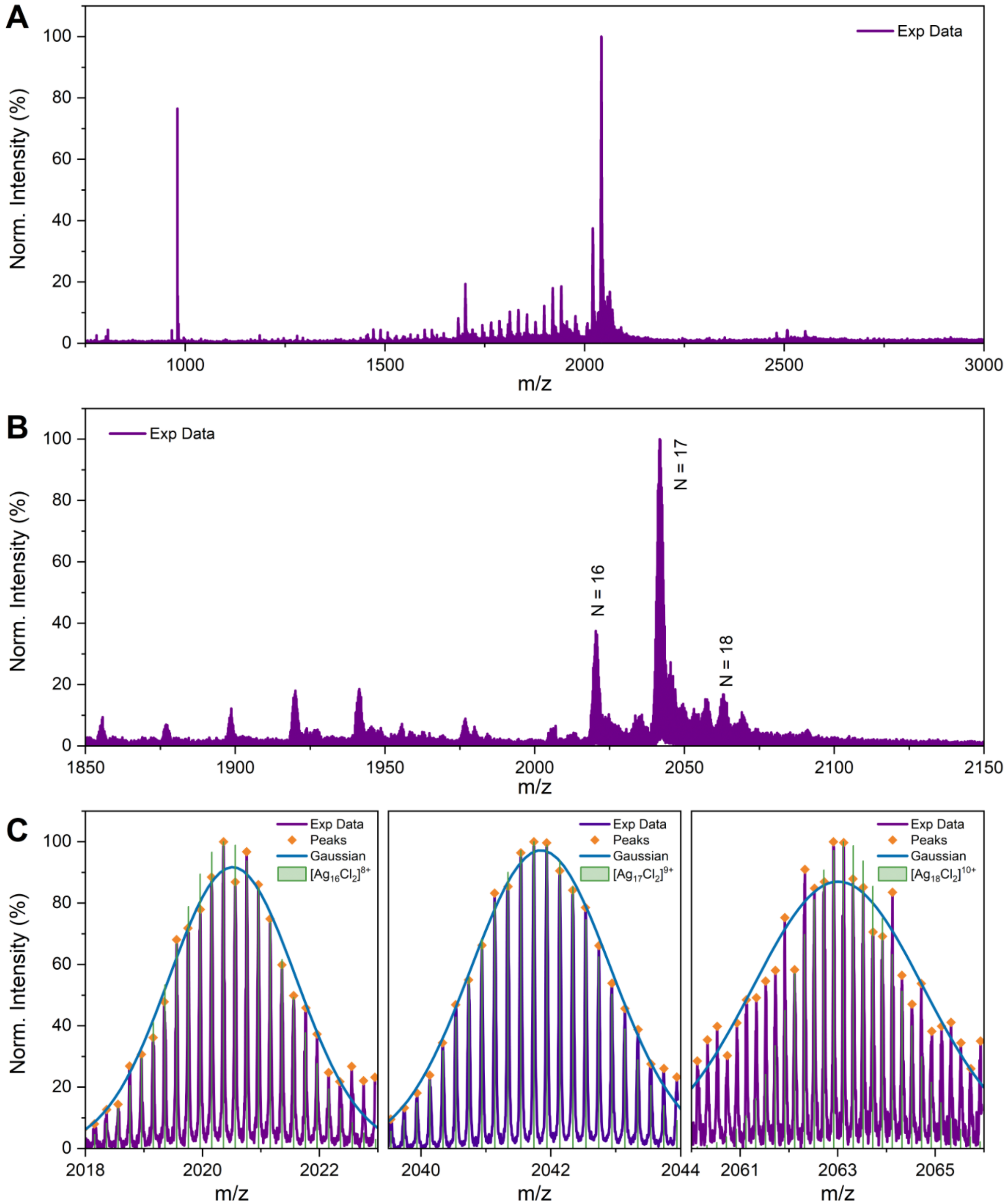

Figure S5: (A) Mass spectra of 2xDNA(T_6)-B-[$Ag_{16}Cl_2$]$^{8+}$ in 10 mM NH_4OAc at room temperature, measured in negative ion mode. (B) Zoomed-in view of z = 5- region. (C) Experimental isotopic distribution with the corresponding Gaussian fit (blue) and the theoretical isotopic distribution (green) for [$Ag_{16}Cl_2$]$^{8+}$(left), [$Ag_{17}Cl_2$]$^{9+}$ (middle) and [$Ag_{18}Cl_2$]$^{10+}$(right) for z=5-. Experimental average masses are listed in Table S1.

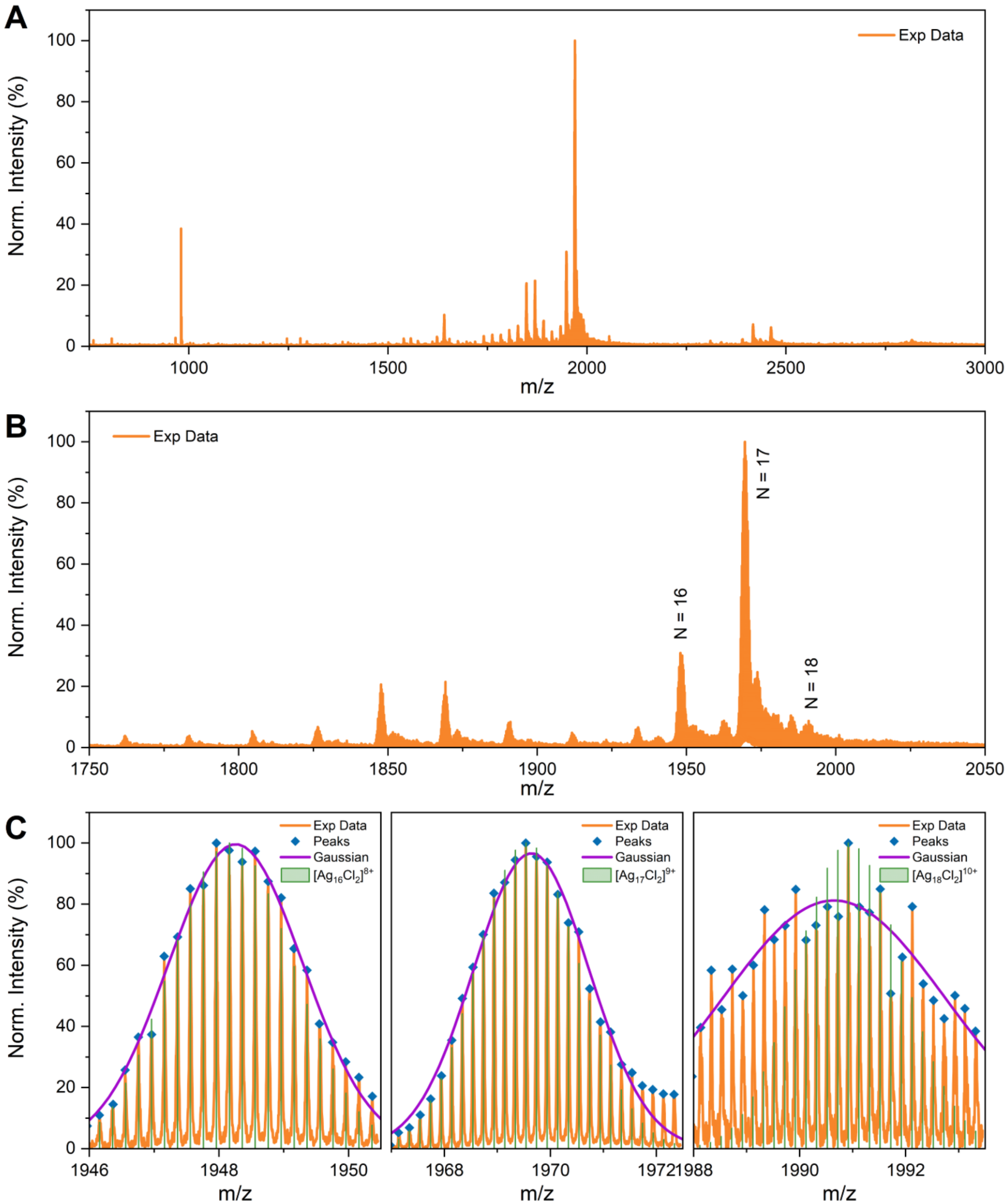

Figure S6: (A) Mass spectra of 2xDNA(TT-B-TT)-$[Ag_{16}Cl_2]^{8+}$ in 10 mM NH_4OAc at room temperature, measured in negative ion mode. (B) Zoomed-in view of z = 5- region. (C) Experimental isotopic distribution with the corresponding Gaussian fit (purple) and the theoretical isotopic distribution (green) for $[Ag_{16}Cl_2]^{8+}$(left), $[Ag_{17}Cl_2]^{9+}$ (middle) and $[Ag_{18}Cl_2]^{10+}$(right) for z=5-. Experimental average masses are listed in Table S1.

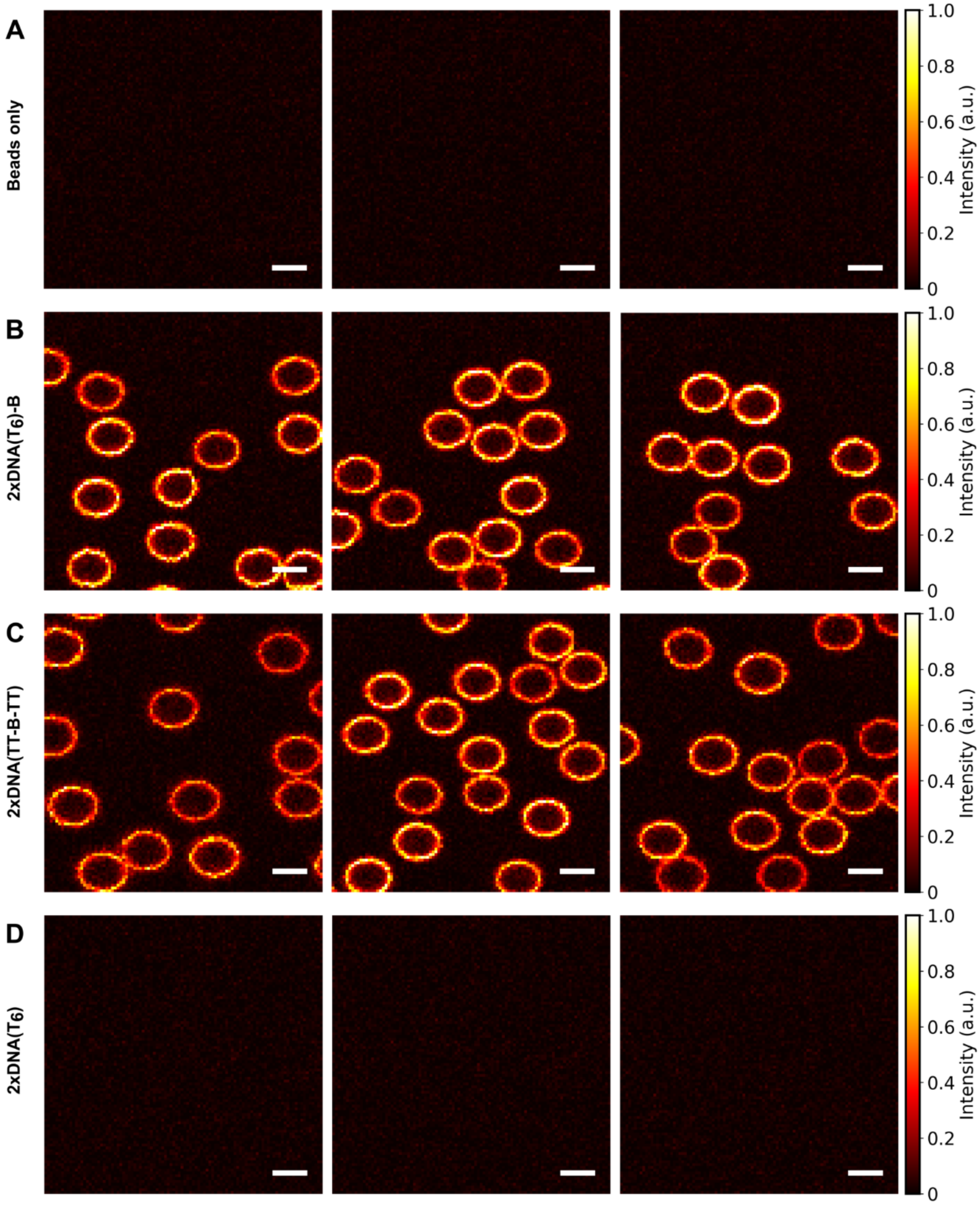

Figure S7: Confocal fluorescence microscopy images of (A) PMMA beads only (See Figure S9 for a representative bright field image) and beads labeled with (B) 2×DNA(T$_6$)-B-[$Ag_{16}Cl_2$]$^{8+}$, (C) 2×DNA(TT-B-TT)- [$Ag_{16}Cl_2$]^{8} and (D) 2×DNA(T$_6$)-[$Ag_{16}Cl_2$]$^{8+}$.

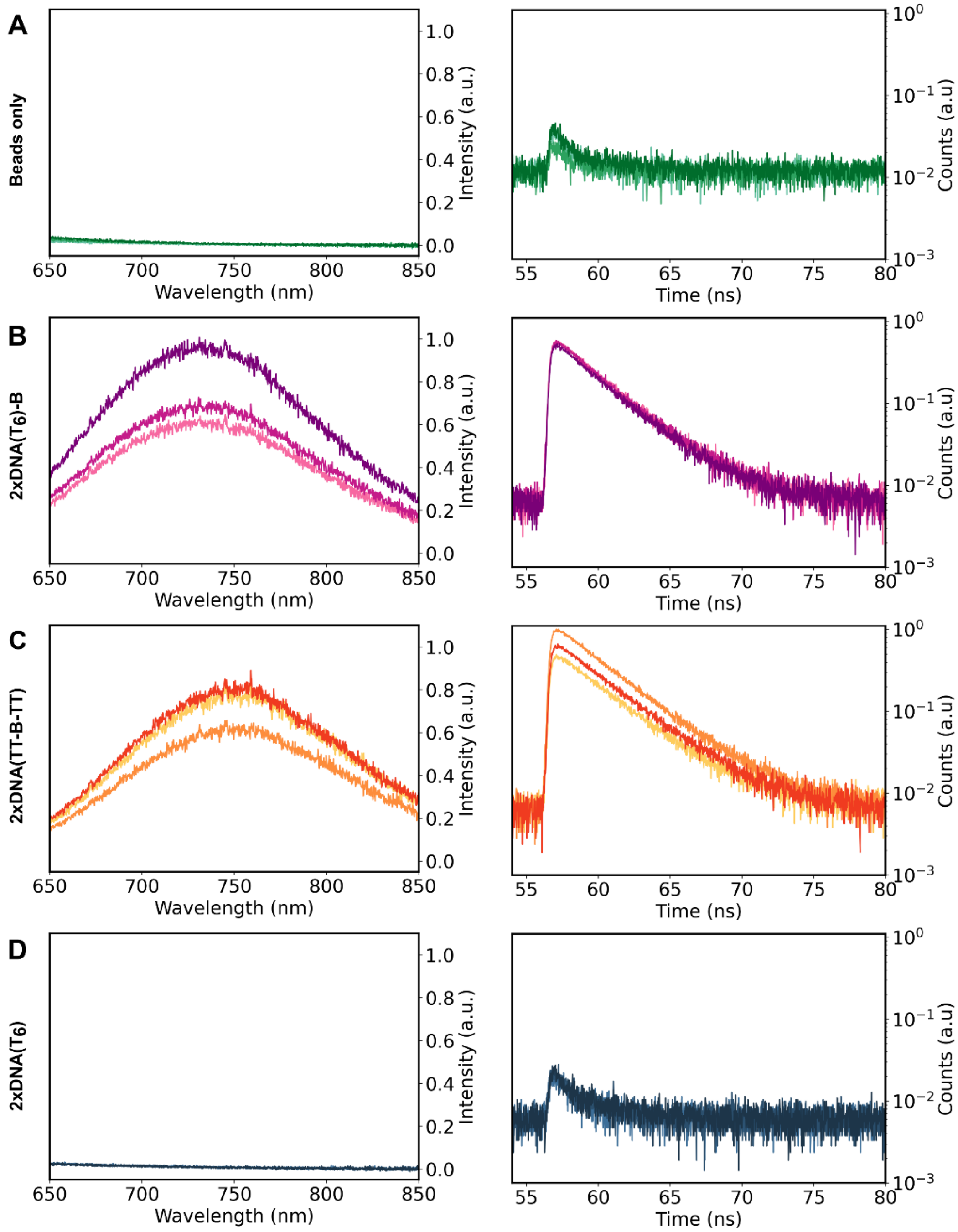

Figure S8: Emission spectra (left) and corresponding decays (right) of the screened beads shown in Figure S7. Beads only (A) exhibited decay times of 1.47, 1.81, and 1.99 ns for the light-green, green, and dark-green spectra, respectively. Beads labeled with 2×DNA(T_6)-B-$[Ag_{16}Cl_2]^{8+}$ (B) showed <τ> decay times of 2.89, 2.88, and 2.89 ns, with emission maxima at 732, 734, and 731 nm for the pink, magenta, and purple spectra, respectively. Beads labeled with 2×DNA(TT-B-TT)- $[Ag_{16}Cl_2]^{8}$ (C) exhibited <τ> decay times of 3.18, 3.18, and 3.17 ns, with emission maxima at 753, 752, and 750 nm for the yellow, orange, and red-orange spectra, respectively. The control beads incubated with 2×DNA(T_6)- $[Ag_{16}Cl_2]^{8}$ (D) showed <τ> decay times of 1.86, 2.37, and 2.35 ns for the light-blue, blue, and dark-blue spectra, respectively. No distinct emission maxima were observed for A and D. The results for D indicate that no significant non-specific binding of the DNA-AgNCs to the beads occurs. The intensity of all spectra were renormalized to the most intense spectrum from B.

Table S2. *Overview of intensity-weighted average lifetime <τ> and corresponding* X^2 *values, amplitudes (*A_i*) and decay times (*τ$_i$*) obtained by fitting the fluorescence decays shown in Figures 3, S4 and S8. The decay curves were fitted using a mono- or bi-exponential reconvolution models. The decays shown in Figure S4 were recorded using the FluoTime 300, whereas those shown in Figure S8 and Figure 3 were measured using our home-build microscope.*

Figure	Decay	〈τ〉	X^2	A_1	τ$_1$	A_2	τ$_2$
S4	2xDNA(T_6)-B	3.43	0.925	9596.4	3.74	5753.0	2.74
S4	2xDNA(TT-B-TT)	3.46	0.901	7723.0	3.66	3392.1	2.89
S8 (A)	Light green	1.47	0.9920	34.7	1.47	-	-
	Green	1.81	0.9912	17.8	1.81	-	-
	Dark green	1.99	1.0226	33.9	1.99	-	-
3 and S8 (B)	Pink	2.89	0.9808	1350.8	2.89	-	-
	Magenta	2.88	1.0570	1342.1	2.88	-	-
	Purple	2.89	1.0934	1256.4	2.89	-	-
3 and S8 (C)	Yellow	3.18	0.9923	1139.1	3.18	-	-
	Orange	3.18	1.0298	2415.5	3.18	-	-
	Orange-red	3.17	0.9808	1982.2	3.17	-	-
S8 (D)	Light blue	1.86	0.9806	25.7	1.86	-	-
	Blue	2.37	0.9147	24.3	2.37	-	-
	Dark blue	2.35	0.8608	22.3	2.35	-	-

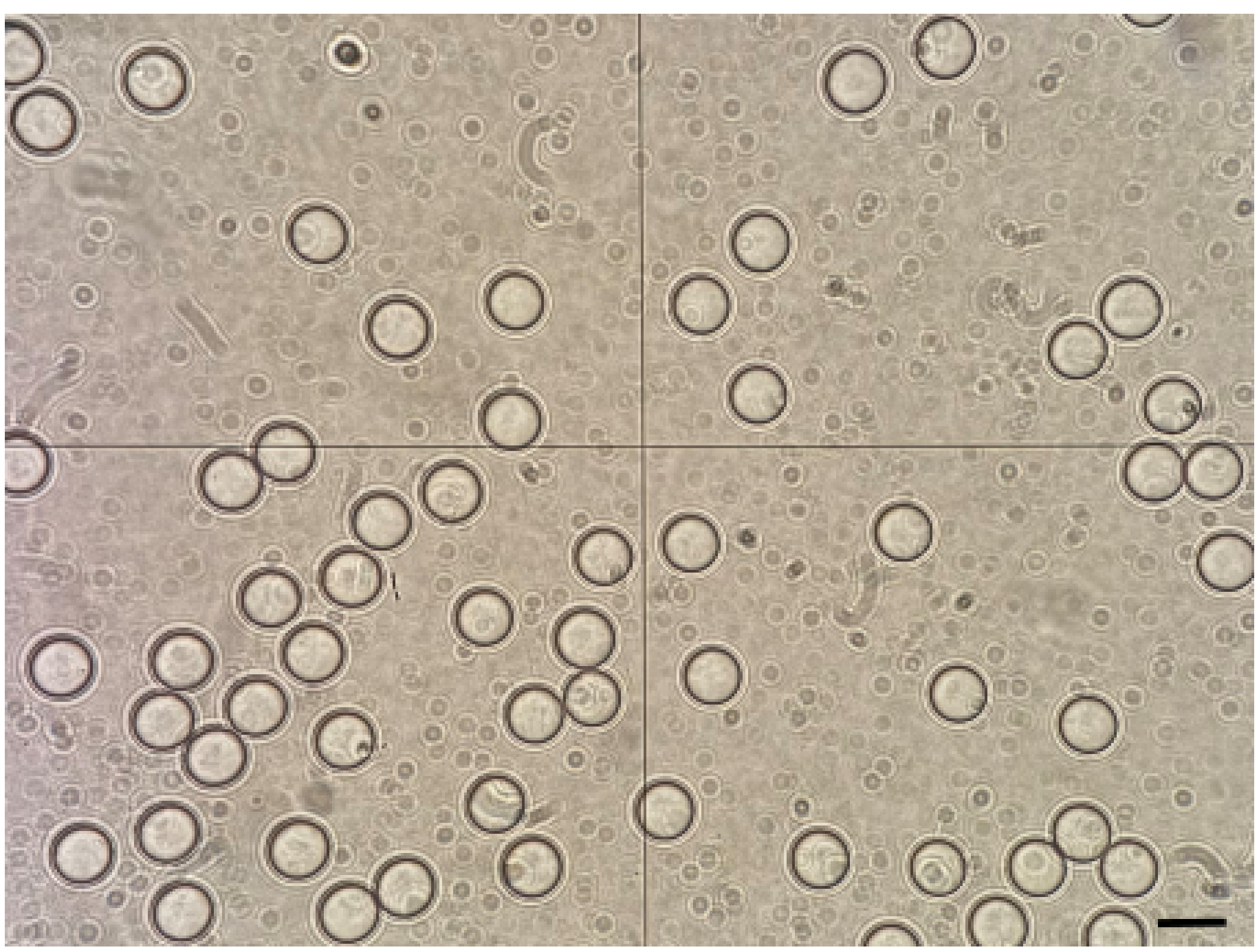

Figure S9: Bright-field image of the PMMA micro-spheres. Scale bar: 10 μm.

References

1. S. A. Bogh, M. R. Carro-Temboury, C. Cerretani, S. M. Swasey, S. M. Copp, E. G. Gwinn and T. Vosch, *Methods and Applications in Fluorescence*, 2018, **6**, 024004.
2. M. Loos, C. Gerber, F. Corona, J. Hollender and H. Singer, *Analytical Chemistry*, 2015, **87**, 5738-5744.
3. M. B. Liisberg, Z. Shakeri Kardar, S. M. Copp, C. Cerretani and T. Vosch, *The Journal of Physical Chemistry Letters*, 2021, **12**, 1150-1154.
